# Bayesian Lasso and multinomial logistic regression on GPU

**DOI:** 10.1371/journal.pone.0180343

**Published:** 2017-06-28

**Authors:** Rok Češnovar, Erik Štrumbelj

**Affiliations:** Faculty of computer and information science, University of Ljubljana, Večna pot 113, 1000, Ljubljana, Slovenia; Mayo Clinic Arizona, UNITED STATES

## Abstract

We describe an efficient Bayesian parallel GPU implementation of two classic statistical models—the Lasso and multinomial logistic regression. We focus on parallelizing the key components: matrix multiplication, matrix inversion, and sampling from the full conditionals. Our GPU implementations of Bayesian Lasso and multinomial logistic regression achieve 100-fold speedups on mid-level and high-end GPUs. Substantial speedups of 25 fold can also be achieved on older and lower end GPUs. Samplers are implemented in OpenCL and can be used on any type of GPU and other types of computational units, thereby being convenient and advantageous in practice compared to related work.

## Introduction

Bayesian methods play an increasingly important role in modern statistics and machine learning due to their robustness and conceptual simplicity. One of the main drawbacks of Bayesian methods is that it is often analytically and computationally difficult to infer from the posterior distribution. We typically resort to structural approximations or computationally intensive sampling-based approximations, in particular, Markov Chain Monte Carlo (MCMC). In this paper, we describe an efficient Bayesian parallel implementation of two classic statistical models—the Lasso (L_1_-regularized regression) and multinomial logistic regression. Our implementation is based on two existing data-augmentation approaches that lead to efficient Gibbs sampling schemes for posterior inference from the two models. We focus on parallelizing the key components: matrix multiplication, matrix inversion, and sampling from the full conditionals. All the methods described in this paper are implemented in the R package bayesCL (https://cran.r-project.org/web/packages/bayesCL/index.html).

Graphics Processing Units (GPUs) have been extensively used for computationally intensive applications since the introduction of CUDA and OpenCL. In essence, a GPU is a massively parallel co-processing unit, with thousands of smaller cores that are optimized to run in parallel. To efficiently run an application on a GPU, we need to split the application into a large number of threads, usually grouped in blocks. The exact number of threads is a trade-off between utilizing all of the many computing cores in a GPU and the cost of creating additional threads. For efficient execution, the threads should require minimal communication, have their memory accesses coalesced and avoid divergent paths of execution. To fully utilize the memory hierarchy of the GPU, threads within blocks should use shared memory whenever possible.

Bayesian statistics on the GPU have already received some attention, both model specific and in general. A GPU implementation for Bayesian mixture models with MCMC is outlined in [1]. The R package *cudaBayesReg* implements the Bayesian multilevel model for the analysis of brain fMRI data [2]. The parallel implementation produces a speed-up of 60 compared to the CPU-only version. A GPU implementation of the Bayesian inference of the Realized Stochastic Volatility Model is proposed in [3]. The GPU implementation produces a speed-up of 17 compared to the CPU version of the algorithm. BioEM [4] is a hybrid CPU-GPU implementation of the Bayesian inference of electron microscopy images. A case-study on population-based Markov chain Monte Carlo methods and Sequential Monte Carlo methods shows GPU implementations achieve speedups from 35- to 500-fold over single-threaded CPU execution [5]. A GPU-accelerated algorithm for Latent Dirichlet Allocation, based on collapsed Gibbs sampling, is proposed in [6]. Terenin et al. [7] proposed a Gibbs-sampling-based GPU implementation of Horseshoe regularized probit regression. They demonstrate speedups on the order of 100s compared to the sequential R version of the algorithm. The work most related to our own is by Beam et al. [8], who proposes a GPU implementation of the Bayesian multinomial logistic regression model with Hamiltonian Monte Carlo. They demonstrate speedups on the order of 10 compared to the R package *glmnet* [9] and speedups of approximately 100-fold for individual key components of HMC (the leapfrog update and gradient calculation). However, we use more efficient Gibbs sampling, and there is no need for leapfrog updates or gradient calculations.

Note that all these related works base their GPU implementations on CUDA, which only supports NVIDIA GPUs. Instead, we use OpenCL, which supports the GPUs of multiple vendors (NVIDIA, AMD/ATI, and Intel) and thus makes our implementations more portable. OpenCL also has better support for Just-in-time compilation, which enables a more thorough approach to run-time optimization for different GPU architectures. OpenCL also supports the execution of the same implementations on massively parallel processors, such as the Xeon Phi, as well as multi-core CPUs for cases when no GPUs are present.

In the remainder of this section, we introduce the Gibbs sampling schemes for the Bayesian Lasso and Bayesian multinomial logistic regression. We proceed by describing our parallelization methodology and empirically evaluating it on two real-world datasets.

### Bayesian Lasso

Let *y* be the *n* x 1 vector of responses, and let *X* be the *n* x *p* matrix of standardized predictors. The classic linear regression model is

$$y_{i}∼N\left(\beta^{T}x_{i},\sigma^{2}\right),$$

where *x*_*i*_ is the column vector representing the i-*th* row of *X*, *σ*^2^ is the unknown variance, and *β* = (*β*_1_, *β*_2_, …, *β*_*p*_)^*T*^ is the set of coefficients to be estimated.

The Lasso is an approach to estimating the coefficients *β* by penalizing the least squares estimate with the regularization term $\lambda \sum_{i=1}^{p}\;\left|\beta_{i}\right|$ for some value of the regularization penalty parameter λ [10]. Due to the *L*_1_-norm penalty, this approach is often referred to as *L*_1_-regularization. Due to the built-in coefficient shrinkage (feature selection), it is particularly useful in cases where the number of predictors is of the same or higher order of magnitude than the number of observations.

From a Bayesian perspective, the Lasso can be interpreted as a least-squares linear regression with independent Laplace priors on the coefficients. We base our Bayesian implementation of the Lasso on the scale-mixture of normals Gibbs sampling scheme by [11]. We place an inverse Gamma prior on the variance *σ*^2^ ∼ Gamma(*a*, *b*) and a Gamma hyperprior on the regularization parameter λ^2^ ∼ Gamma(*r*, *δ*) (see [11] for details). This results in the following full conditionals and algorithm:

precompute *X*^*T*^ *X* and $\tilde{y}=y-\bar{y}$

**foreach** *Gibbs sampling iteration* **do**

 $D_{\tau }=\text{diag}\left(\tau_{1}^{2},\ldots ,\tau_{p}^{2}\right)$

 $\Sigma =X^{T}X+D_{\tau }^{-1}$

 $\beta_{\mu }=\Sigma^{-1}X^{T}\;\tilde{y}$

 *β*|*σ*^2^, *τ*^2^ ∼ *N*(*β*_*μ*_, *σ*^2^Σ^−1^)

 $\tau_{i}^{2}|\beta$, *σ*^2^, $\lambda^{2}∼\text{Inv-}N\left(\sqrt{\frac{\lambda^{2}\sigma^{2}}{\beta_{i}^{2}}},\lambda^{2}\right)$

 $q=\frac{1}{2}{\left(\tilde{y}-\beta^{T}X\right)}^{T}\left(\tilde{y}-\beta^{T}X\right)+\frac{1}{2}\beta^{T}D_{\tau }^{-1}\beta$

 *σ*^2^|*β*, $\tau^{2}∼\text{Inv-Gamma}\left(\frac{n-1+p}{2},q\right)$

 λ^2^|*τ*^2^, $\delta ∼\text{Gamma}\left(p+r,\sum \;\frac{\tau_{j}^{2}}{2}+\delta \right)$

**end**

This variant of the Gibbs sampler for the Bayesian Lasso already has a CPU implementation in the R package *monomvn* [12]. We use it as a baseline for comparison in the empirical evaluation. Note that the package also supports other types of regularization (ridge and Horseshoe) and Reversible Jump MCMC for model selection.

### Bayesian multinomial logistic regression

Let *Y* be the *n* x *k* matrix of responses, where *n* is the number of observations and *k* is the number of response categories. Let *y*_*ij*_ = *Y*_*ij*_ be the count of responses in category *j* for observation *i*, and $n_{i}=\sum_{j=1}^{p}\;y_{ij}$. Again, let *X* be the *n* x *p* matrix of predictors. The classic multinomial logistic regression model is

$$\left(y_{i1},\ldots ,y_{ik}\right)∼\text{Multinomial}\;(n_{i},\sigma \left({\left(\beta_{1}^{T}x_{i},\beta_{2}^{T}x_{i},\ldots ,\beta_{k}^{T}x_{i}\right)}^{T}\right))$$

where *x*_*i*_ is the i-*th* row of *X*, *β*_*j*_ is the *j*-th column of the *p* x *k* coefficient matrix *β*, and $\sigma {\left(z\right)}_{i}=\frac{e^{z_{i}}}{\sum_{j=1}^{k}\;e^{z_{j}}}$ is the vector softmax function. For identifiability, *β*_*k*_ = 0_*p*_.

Typically, Laplace approximation [13, p. 213] or the Metropolis algorithm [13, p. 541] is used to infer from logistic models. Recently, Polson et al. [14] proposed a data-augmentation scheme that leads to a simple Gibbs sampler for Bayesian logistic regression. This data-augmentation scheme is more efficient than Metropolis samplers and other data approaches (see [14] for details) and easily extends to other categorical and count models such negative binomial regression and our case of multinomial logistic regression. We place independent normal priors of the coefficients *β*_*j*_ ∼ *N*_*p*_(*β*_0*j*_, Σ_0*j*_). This results in the following Gibbs sampling algorithm for inferring from the posterior:

precompute $\kappa_{ij}=y_{ij}-\frac{1}{2}n_{i}$

**foreach** *Gibbs sampling iteration* **do**

 **foreach** category *j* = 1..(*k* − 1) **do**

  *D*_*ω*_ = diag(*ω*_1*j*_, …, *ω*_*nj*_)

  $\Sigma ={\left(X^{T}D_{\omega }X+\Sigma_{0j}^{-1}\mu_{0}\right)}^{-1}$

  $\beta_{\mu }=\Sigma \left(X^{T}\kappa +\Sigma_{0j}^{-1}\mu_{0j}\right)$

  *β*_*j*_|*w* ∼ N_*p*_(*β*_*μ*_, Σ)

  *ω*_*ij*_|*β*_*j*_ ∼ Polya-Gamma(*n*_*i*_, *β*_*j*_ *x*_*i*_), *i* = 1..*n*

 **end**

**end**

This variant of the Gibbs sampler for the Bayesian multinomial logistic regression also has a CPU implementation in the R package *bayesLogit* [15]. We use it as a baseline for comparison in the empirical evaluation.

#### Polya-Gamma distribution

The Polya-Gamma (PG) distribution Polya-Gamma(*h*, *z*) is a two-parameter continuous distribution (*h* > 0, *z* ∈ *R*) (for a definition of its density, see [14]).

The PG distribution is not easy to sample from and can produce a performance bottleneck, in particular when *n*_*i*_ are large. In this paper, we are interested in sampling from Polya-Gamma(*h*, *z*) when *h* is an integer. An important property of the PG distribution is that if both *X* ∼ PG(*h*_*x*_, *z*) and *Y* ∼ Polya-Gamma(*h*_*y*_, *z*), then *X* + *Y* is distributed as Polya-Gamma(*h*_*x*_ + *h*_*y*_, *z*). Therefore, for an integer *h*, a random variate from PG(*h*, *z*) can be generated using *h* random variates from Polya-Gamma(1, *z*). This allows us to focus on sampling from PG(1, *z*).

Our parallel algorithm is based on the algorithm from [14], which is based on sampling from the (exponentially tilted) Jacobi distribution J*(1,z) [16] and the relationship whereby if *X* ∼ J*(1,z/2), then *Y* = *X*/4 ∼ PG(1, *z*). Therefore, our sampling problem reduces to sampling from J*(1,z). The density *f*(*x*) of a J*(1,z) distributed random variable contains an infinite sum and can only be approximated (the same obviously holds for the density of PG(1,z)), which makes sampling difficult. However, it has an alternating series representation

$$f\left(x\right)=\sum_{i=0}^{\infty }{\left(-1\right)}^{i}cosh\left(z\right)\exp\;\{-\frac{z^{2}x}{2}\}\;a_{i}\left(x\right),$$

$$a_{i}\left(x\right)=\{\begin{array}{cc} \pi \left(i+1/2\right){\left(\frac{2}{\pi x}\right)}^{3/2}\exp\;\{-\frac{2{\left(i+1/2\right)}^{2}}{x}\} & 0<x<t\\ \pi \left(i+1/2\right)\exp\;\{-\frac{2{\left(i+1/2\right)}^{2}\pi^{2}}{2}x\} & x>t, \end{array}\}$$

where the optimal choice of *t*, where this two-part bounding curve fits *f*(*x*) most tightly, is near 0.64 (see [16] for details).

The partial sums of the series $S_{i}\left(x\right)=\sum_{j=0}^{i}{\left(-1\right)}^{j}a_{j}\left(x\right)$ satisfy

$$S_{0}\left(x\right)>S_{2}\left(x\right)>\cdots >f\left(x\right)>\cdots \;S_{3}\left(x\right)>S_{1}\left(x\right),$$

which facilitates a modified rejection algorithm for sampling from *f*(*x*). The problem that *f*(*x*) cannot be evaluated is circumvented by iteratively calculating the partial sums and stopping early using the above property of *S*_*i*_(*x*). Given an upper hull (envelope) function *g*(*x*) > *f*(*x*), we can sample from *f*(*x*) by sampling from *g*(*x*) and accepting each sample *x*_0_ with probability *f*(*x*_0_)/*g*(*x*_0_), which can be done by sampling *U* ∼ *U*(0, *g*(*x*_0_)) and checking *U* < *f*(*x*_0_). Due to the property of the partial sums, we know that all odd terms are less than *f*(*x*); therefore, if *U* < *S*_*i*_(*x*_0_) for odd *i*, then we can deduce that *U* < *f*(*x*) and accept the sample. Similarly, if *U* > *S*_*i*_(*x*_0_) for even *i*, then we can deduce *U* > *f*(*x*) and reject the sample.

The first partial sum *S*_0_(*x*) is a natural (and efficient [14]) choice for the upper hull function *g*(*x*), and it can be sampled from a mixture of a truncated inverse-Gaussian distribution and a truncated exponential distribution

$$X∼\left\{ \begin{array}{cc} {\text{IG}(|z|}^{-1},1)I_{\left(0,t\right]} & \text{with}\;\text{prob.}\;p\;\text{see}\;[14]\\ \text{Exp}\left(-z^{2}/2+\pi^{2}/8\right)I_{\left(t,\infty \right]} & \text{with}\;\text{prob.}1-p. \end{array}\right.$$

Left-truncated exponential variates can be generated trivially by translating and scaling a Exp(1) variate. Inverse-Gaussian variates are sampled using the method of [17], which is based on Exp(1) and standard normal N(0, 1) variates. For further details on the above algorithm for sampling from J*(1,z) and therefore PG(1, *z*), see [14]. Note that other, faster approximate approaches for sampling from PG have been developed [18] but are unstable for larger *h*.

## Parallelization

The parallel implementations that are described below are a part of the bayesCL package for R. Input checking is mostly implemented in R, while the computations are implemented in C and OpenCL. OpenCL was used to support GPUs of all vendors.

### Matrix multiplication and matrix-vector multiplication

The common case of matrix multiplication is the case with square matrices or non-square matrices with dimensions of the same order. These cases of matrix multiplication were implemented as advised in GPU parallel programming guides and papers on the optimization of matrix multiplication [19–21]. When not stated otherwise, each created thread computes a single value of the resulting matrix. All the matrix multiplications are performed by tiling the input matrices to use shared memory. No architecture-specific optimization is used to support a wider range of GPUs. When the input matrices are large (tens of millions of values in the resulting matrix) in these common cases of matrix multiplication, it is sometimes useful to have single threads compute multiple values of the resulting matrix. The exact number of values that a single thread calculates depends on the size of the input matrix and the GPU used. This is used in the matrix multiplication of the inverse matrix.

In Bayesian computation, matrix multiplications of dimensions *N* × *M* times *M* × *N* arise, where *M* is orders of magnitude larger than *N*. The resulting matrix is therefore of the size *N* × *N*. If we were to use the parallelization discussed in the previous paragraph, we would create *N* × *N* threads, and each thread would calculate the product of *M* elements and sum these products. This means that each thread has a substantial workload (*M* can be larger than 500,000), while the overall number of threads is small (1000–3500). In such cases, we instead split the calculation into two parts/kernels. In the first part, we create *T* × *N* × *N* threads in such a way that each thread computes 1/*T*-th of the products and creates a partial sum. In the second step, we create *N* × *N* threads, where each thread calculates the sum of all *T* partial sums for one element of the resulting matrix.

### Bayesian Lasso

The bottlenecks in each pass of the main loop in the Bayesian Lasso algorithm are the calculations of the inverse of the covariance matrix, sampling the coefficients *β* from the full conditional, and matrix operations needed to compute *β*_*μ*_. These parts are the main focus of this implementation and are explained in detail in the following subsections. All other parts of the main loop are also executed on the GPU to avoid unnecessary data transfers between the CPU and GPU. Their execution times on the CPU are negligible, and their parallel implementations are trivial and thus omitted.

The matrix inverse is calculated by multiplying the inverse of the lower triangular Cholesky decomposition of the input matrix and its transpose. The Cholesky decomposition is also the main step in sampling *β*. The focus of our parallelization of the Bayesian Lasso algorithm was therefore based on three steps: the Cholesky decomposition, the inversion of a lower triangular matrix, and matrix multiplication.

### Data transfers

In the initialization phase of the algorithm, the covariance matrix is calculated on the GPU. The input matrix to this calculation is the X matrix with *M* × *N* values. This is the only non-negligible data transfer in the initialization phase. In the main loop, *M* random seeds used for generating random values are transferred to the GPU, and *M* resulting *β* values are transferred from the GPU to global memory on each pass. Because *M* usually numbers from a few tens to a few thousand, these data transfers do not represent a large enough overhead to suppress the speedup of the GPU execution and are also negligible in terms of the overall execution time.

### Cholesky decomposition

**Fig 1:**
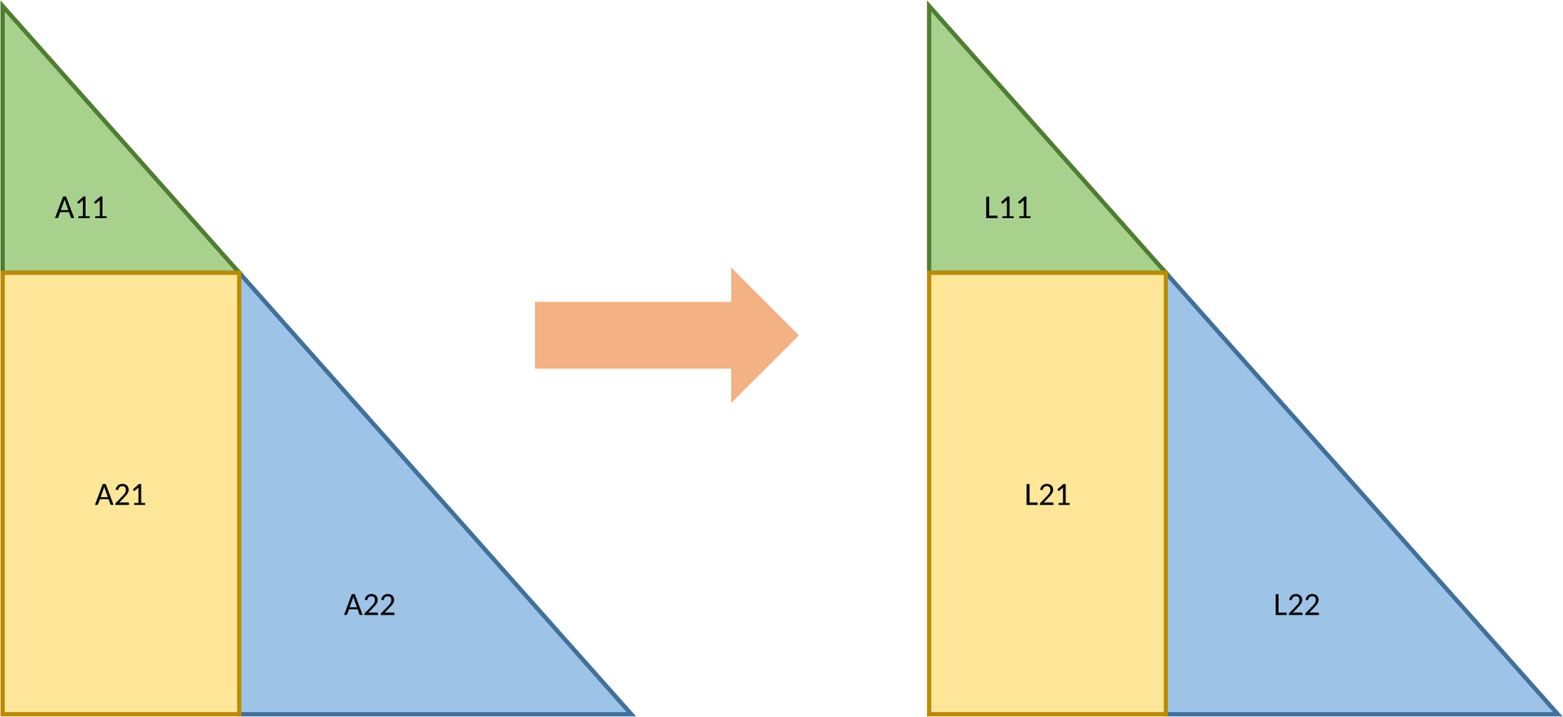
A step of the blocked Cholesky decomposition.

For the Cholesky decomposition of a squared matrix A, we implemented a variation of the blocked version of the algorithm, proposed in [22]. The input matrix is first split into parts as shown in Fig 1. The size of the block A_11_ depends on both the properties of the GPU and the input matrix size. In the first step, we calculate L_11_, which is the Cholesky decomposition of A_11_ and the inverse that is used in the next steps. In this first step, we create a single block of threads with the number of threads equal to the dimension of L_11_. The decomposition and the inverse A_11_ are calculated in local memory. Sequential parts of the algorithm are calculated by a single thread in the thread block. The update steps are executed in parallel with a local synchronization barrier after each parallel execution. The second and third steps of the Cholesky Decomposition are to calculate L_21_ and L_22_ as follows:

$$L_{21}=A_{21}{\left(L_{11}^{T}\right)}^{-1}$$

$$L_{22}=A_{22}-L_{21}{\left(L_{21}\right)}^{T}.$$

Matrix multiplication is again implemented in such a way that each thread computes a single value of the resulting matrix and that the threads use shared memory. After these three steps are completed, we obtain a partial result of the final Cholesky decomposition, as L_11_ and L_21_ can be copied to the resulting matrix. The L_22_ block is again split as shown in Fig 1, and the steps are repeated. When the size of L_22_ is smaller than or equal to the block size of A_11_, only the first step is executed, and the calculation is completed.

### Matrix inversion

**Fig 2:**
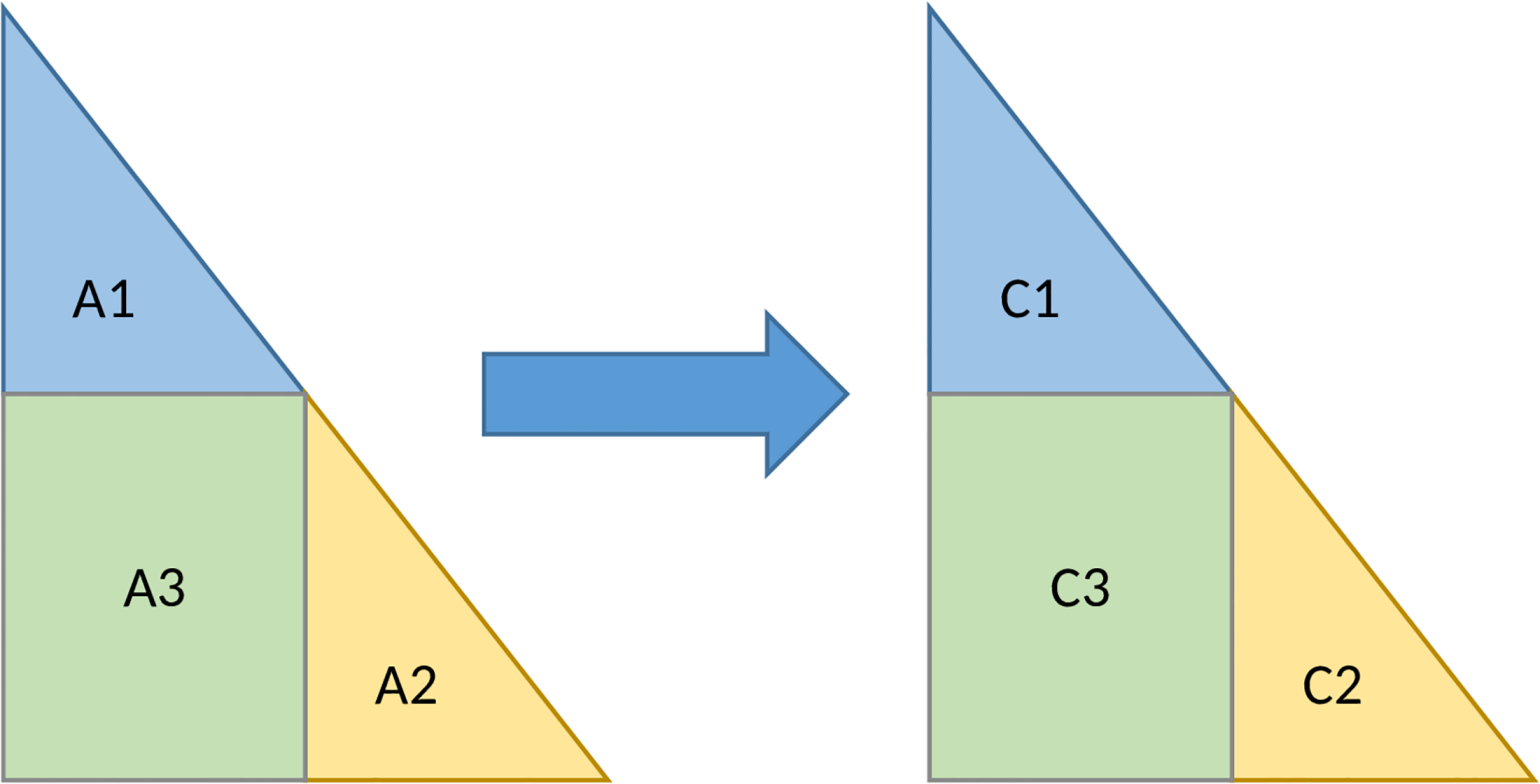
The split of the lower triangular matrix inverse.

The first step of the matrix inversion is the Cholesky decomposition of the input matrix. This is followed by the computation of the inverse of the resulting lower triangular matrix and the multiplication of the inverse with its transpose. The Cholesky decomposition and matrix multiplications are implemented as discussed in the previous subsections. The basic CPU implementation of the inversion of the lower triangular matrices is not suitable for achieving efficient implementation on a GPU. To better utilize the performance of the GPU, the solution proposed in [23] is used. Their basic idea is to split the matrix into blocks, as shown in Fig 2.

First, we calculate the inverses of the smaller matrices A_1_ and A_2_. This is done in parallel, with each smaller matrix assigned to one thread. The remaining part of the inverse is computed using C_3_ = −C_2_A_3_C_1_. These are again matrix multiplication operations that are implemented as previously discussed.

**Fig 3:**
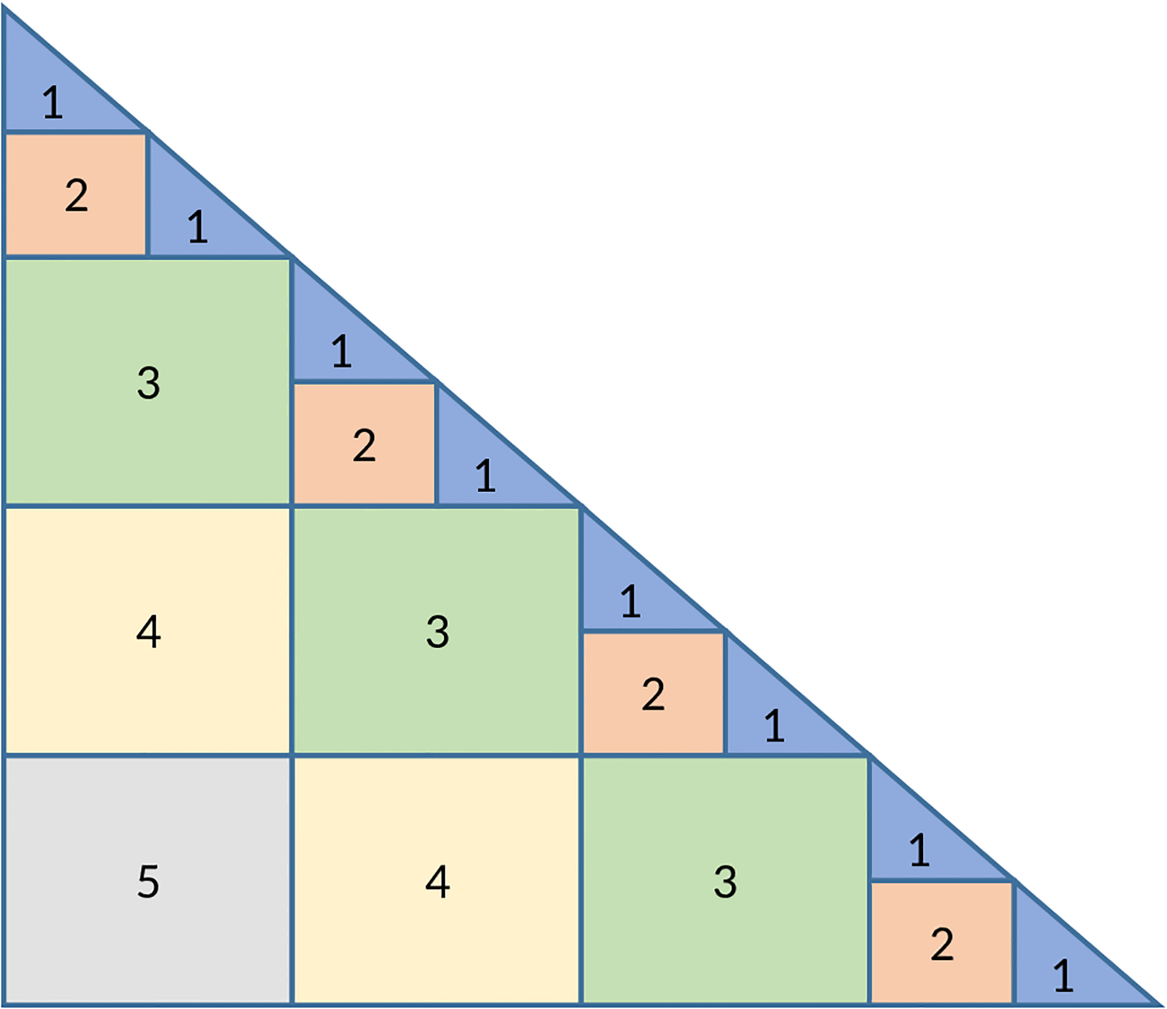
A step of the blocked Cholesky decomposition.

To better utilize the GPU, the matrix should be split into more than two blocks. The implementation supports numbers of blocks that are a power of 2, where the exact number used depends on the properties of the GPU and the input size problem. An example of the execution when the number of blocks is 8 is given in Fig 3.

### Multinomial logistic regression

The algorithm for the multinomial logistic regression is parallelized in full. The main bottlenecks are the sampling from the PG distribution, the Cholesky decomposition, matrix inversions and matrix multiplications. Other parts of the algorithm (summation, copying matrices, etc.) are also executed on the GPU. The speed-up of the parallel execution of these smaller parts on the GPU is not significant, but it eliminates the need to transfer the intermediate results to the CPU and back to the GPU. The parallelization of the aforementioned bottlenecks is described in the following subsections.

#### Sampling from the Polya-Gamma distribution

The basic case of sampling from the distribution is drawing *r* samples from PG(*h*, *z*). We focus on a more generalized problem of performing *l* simultaneous samplings, each with different *r*_*i*_, *h*_*i*_, and *z*_*i*_. The total number of samples to be drawn is $R=\Sigma_{i=1}^{l}r_{i}$.

The naive approach would be to split the problem such that each sampling *i* from PG(*h*_*i*_, *z*_*i*_) is a task. In this way, each task is independent, therefore requiring no communication. However, we may face the problem of uneven workloads because threads that would perform draws with large *h*_*i*_ would have a substantially higher workload than would threads with small *h*_*i*_. Furthermore, with small *R*, the low number of threads would not fully utilize the GPU performance.

To address these drawbacks, we define a task to be a single draw from PG(1, *z*_*i*_). This makes tasks more homogeneous and the workload of the threads more even, as the computational complexity of PG(1, *z*_*i*_) is practically independent of *z*_*i*_. The number of tasks in this parallelization is $R_{\text{task}}=\Sigma_{i=1}^{l}r_{i}h_{i}$. Note that *R*_task_ > *R* if there are *h*_*i*_ > 1; therefore, we are able to better utilize the GPU when *R* is small. The final step is to calculate the sum of *h*_*i*_ samples from PG(1, *z*_*i*_) to calculate the PG(*h*_*i*_, *z*_*i*_) draws, as explained in the PG section in the Introduction. When drawing from PG(*h*, *z*) is used as a standalone process, the final step is performed on the CPU. When it is used as a step in the multinomial logistic regression model, the sums are calculated on the GPU, therein using *r*_*i*_ threads, where each thread calculates a single sum of *h*_*i*_ draws from PG(1, *z*_*i*_).

The homogeneity of the tasks also gives us the ability to easily group them into larger homogeneous workloads. Each thread performs batches of *B* draws from PG(1, *z*_*i*_) instead of single draws. This is useful when searching for the optimal point in the aforementioned trade-off, for example, when *R*_task_ is much greater than the number of execution units *C* on the GPU. If each thread executes a single draw, the benefits of parallelization would become small, as (*R*_task_ − *C*) threads would be assigned to the queue. The cost of creating tasks would therefore outweigh these benefits. In cases where *C* > *R*_task_, we could define *B* = 1, utilizing all the execution units. With the ability to easily fine-tune *B*, we can obtain the best performance from the GPUs of different vendors and under different architectures. Note that parallelization within the execution of single draws from *PG*(1, *z*_*i*_) is not feasible, as these parallel tasks would require substantial communication, thereby outweighing the benefits of parallel execution.

The algorithm for sampling from PG(1, *z*) requires random variates from the distributions *U*(0, 1), *N*(0, 1), and *Exp*(1). We implemented the XORShift128 [24] uniform random number generator, as this generator is one of the fastest on the GPU and possesses satisfactory statistical properties [25, 26]. Unit uniform variates *U*(0, 1) are generated trivially by dividing the generated number by the maximum possible uniform random number. Standard normal variates *N*(0, 1) are generated using *U*(0, 1) variates and the Box-Mueller transform [27]. Exponential variates *Exp*(1) are generated using the *U*(0, 1) variates and the inverse transform method.

#### Data transfers

As noted previously, the whole multinomial logistic regression algorithm is executed on the GPU; therefore, the majority of data transfers are executed only once in the initial phase of the algorithm. Compared to the latter execution of the burn and sampling phase, these data transfers are negligible. During the burn and sampling phase, only the *β* samples are transferred back to the CPU. These transfers are more efficient if all the samples for all sampling steps are transferred after the sampling phase, but this would require us to store all the samples on the GPU; this would not be feasible on low-end GPUs with low amounts of global memory on the GPU. The only data that are transferred to the GPU are new random seeds for the PG sampling phase. Both of these transfers are negligible in terms of their execution time compared to the execution times of other steps.

### Parameters of the parallel implementation

Previous sections noted parameters of the implementation that can be manually tuned for the target GPU. The list of the parameters that were manually tuned for each GPU are given in Tables 1 and 2. We focus on the parameters of the bottlenecks of both implementations. Note that all parameters are positive and integers.

**Table 1:** Implementation parameters for the multinomial logistic regression.

Name	Description
PGbatch	The number of Polya-Gamma samplings for each thread
PGlocal	Threads per block for the Polya-Gamma sampling
MMlocal	Threads per block for the matrix mult. in the logistic regression
MMT	T for the matrix multiplication in the logistic regression
MVT	T for the matrix-vector multiplication in the logistic regression

**Table 2:** Implementation parameters for the Bayesian Lasso.

Name	Description
CholBlock	The size of the block A_11_ in the Cholesky decomposition
MInvBlocks	The number of blocks for the first step of the matrix inversion
MMLocal	Threads per block for the matrix mult. in the inversion
MMWPT	The number of resulting matrix values assigned to a thread

The number of blocks for the fist step of the matrix inversion is determined with M2^−*a*^, where M is the matrix dimension and *a* = MInvBlocks.

## Empirical evaluation

### Data

The data used in the empirical evaluations are motivated by real-world problems. The data are available for download S1 Data.

For the Lasso, we use data from environmental modeling, more precisely, pollutant concentration prediction. The data set consists of 425 observations (days) of 71 predictors (meteorological measurements, meteorological model forecasts, and pollutant concentration data). The goal is to predict the response variable—the next day’s tropospheric ozone concentration. In daily pollutant concentration prediction, it is common to have 100s of observations and 100s or even 1000s of predictors, in particular, if pairwise interactions are considered. This makes regularized regression very suitable for this task.

For the multinomial regression, we use data from a common problem in geography—landscape classification. This problem is relevant for both geographical and economic reasons. In landscape classification, it is very common to have 100,000s of observations and a few orders of magnitude fewer predictors. The data set consists of 506,450 observations (geographical units) of 56 predictors (rainfall and temperature regimen, elevation, rock type, etc.). The goal is to model the relationship between the predictors and the 9 predetermined land types to automatically classify new geographical units.

### Evaluation procedure and hardware

Our goal was to investigate both the maximum speedup and how the speedup depends on the number of predictors for the Lasso and the number of observations for the multinomial logistic regression. For this purpose, we created several data sets from the original data sets with varying numbers of predictors (observations). These data sets were obtained by sampling from the predictors (observations) of the original data set with replacement. All reported measurement points for Lasso are medians of 3 repetitions of 50 iterations of Gibbs sampling and medians of 3 repetitions of 100 iterations of Gibbs sampling for the multinomial logistic regression. All the reported measurements for sampling from PG are medians of 500 repetitions.

We used four different hardware configurations (see Table 3). Note that all speedups are relative to the CPU-only version of the algorithm running on a hardware configuration consisting of an Intel i5-6600K CPU running at 3.50 GHz and 32 GB of DDR4 RAM with a 2400 MHz clock frequency. The execution time for the baseline packages (BayesLogit and monomvn) was measured using the microbenchmark R package [28] because the aforementioned packages do not measure their execution time. The execution time for our implementation was measured in C and was returned to R as one of the resulting vectors. The time spent on passing the input from R to C and the output from C to R is therefore not included in our measurements. Because this time is negligible in terms of the overall execution time, this does not affect the speedups presented in this section.

**Table 3:** The list of hardware configurations.

Name	GPU	CPU
GTX1070	NVIDIA GTX 1070	Intel i5-6600K @ 3.50 GHz
GTX760	NVIDIA GTX 760	Intel i7-4790 @ 3.6 GHz
HD5750	ATI Radeon HD 5750	Intel i7-4790 @ 3.6 GHz
TeslaK20m	Tesla K20m	Intel Xeon E5-2620 @ 2 GHz

Table 4 shows the values used in the empirical evaluations for all the tunable parameters discussed in the previous section. These values were obtained empirically by measuring the execution times for different input sizes on all four hardware configurations.

**Table 4:** Values of the tunable parameters in the experimental evaluation.

Name	GTX1070	GTX760	HD7570	K20m
PGbatch	35	20	25	30
PGlocal	64	256	128	64
MMlocal	32	16	16	32
MMT	256	128	256	256
MVT	128	32	32	128
CholBlock	80	40	64	45
MInvBlocks	5	4	4	5
MMLocal	28	32	32	16
MMWPT	7	4	4	4

### Results

**Fig 4:**
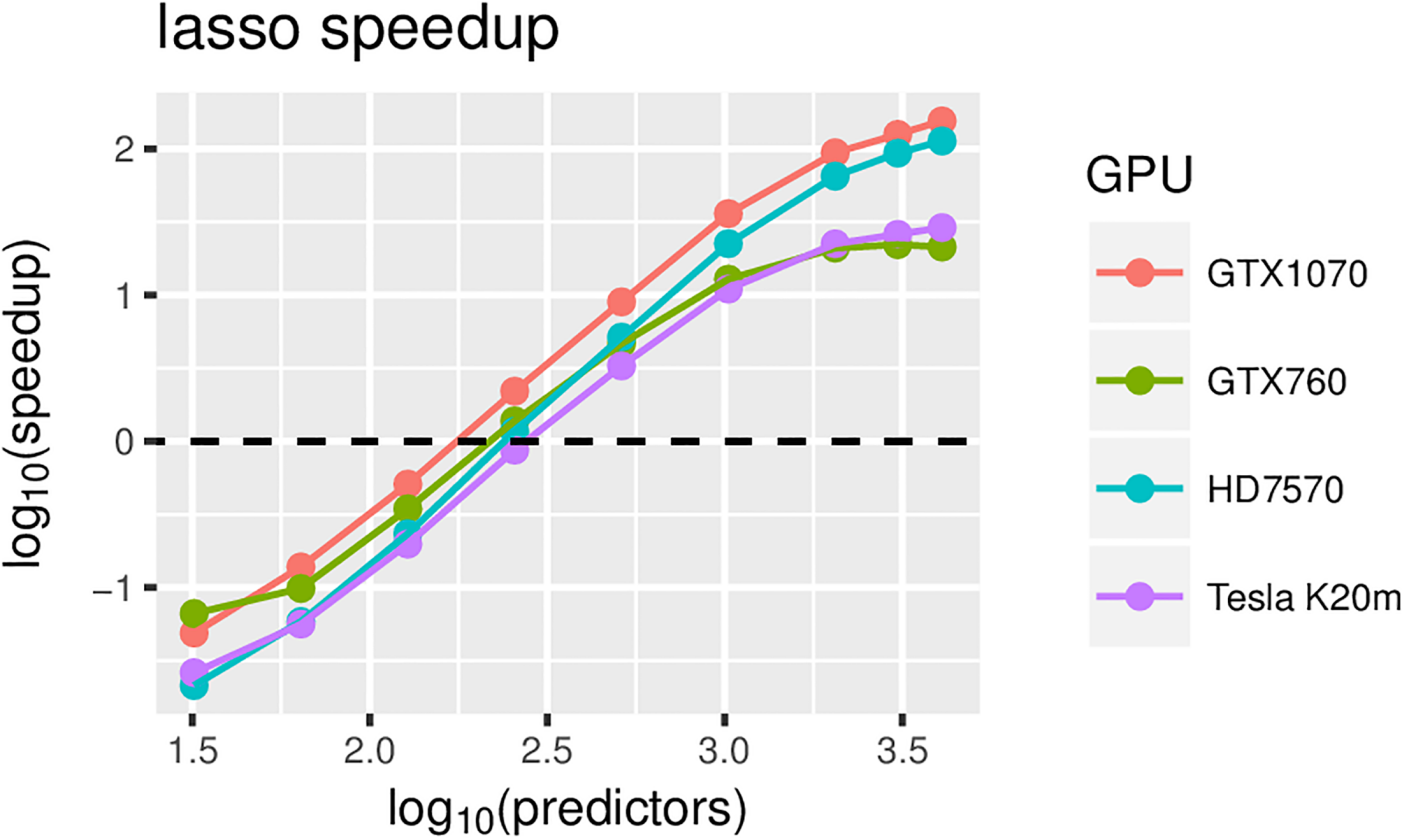
Lasso speedup relative to CPU-only version (on Intel i5-6600K @ 3.50 GHz) for different GPUs and an increasing number of predictors.

**Fig 5:**
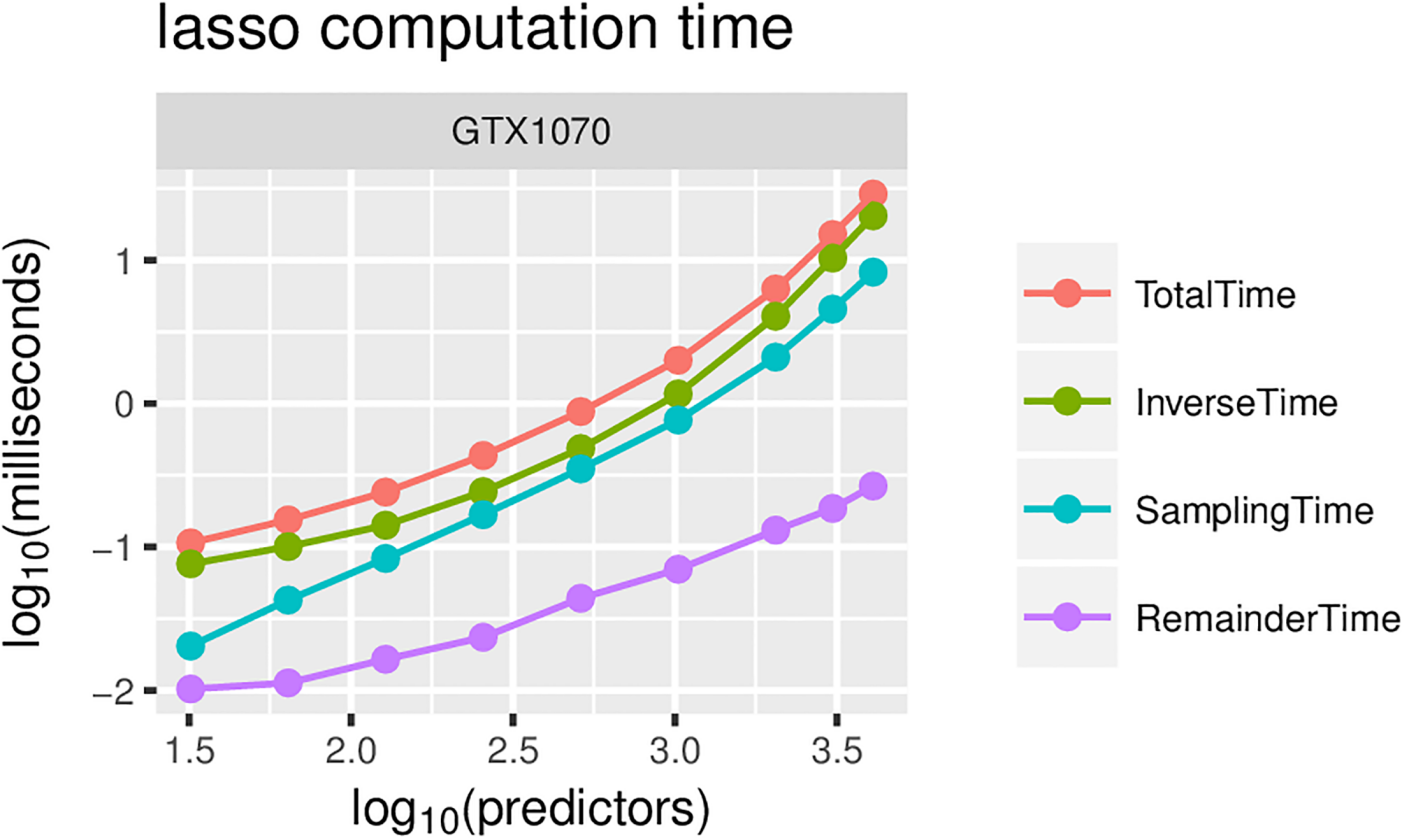
Lasso computation times for GTX1070, broken down by key algorithm components. The results for other GPUs are very similar, and thus, we omit them.

The empirical results show that for Bayesian Lasso, speedups of 100 fold can be achieved for 2000–3000 predictors under high-end hardware configurations (see Fig 4), while maximum speedups of 20 fold can be achieved for low-end and mid-range GPUs. The maximum measured speedup was 150 fold on the GTX1070 GPU. The computation with the GPU version is dominated by the time needed to calculate the inverse and to sample from the multivariate normal, where Cholesky decomposition is calculated (see Fig 5). The difference between these two components is fairly constant—the former represents approximately 70% of the computation time, while the latter represents approximately 30%. The time spent on other parts of the algorithm becomes negligible.

**Fig 6:**
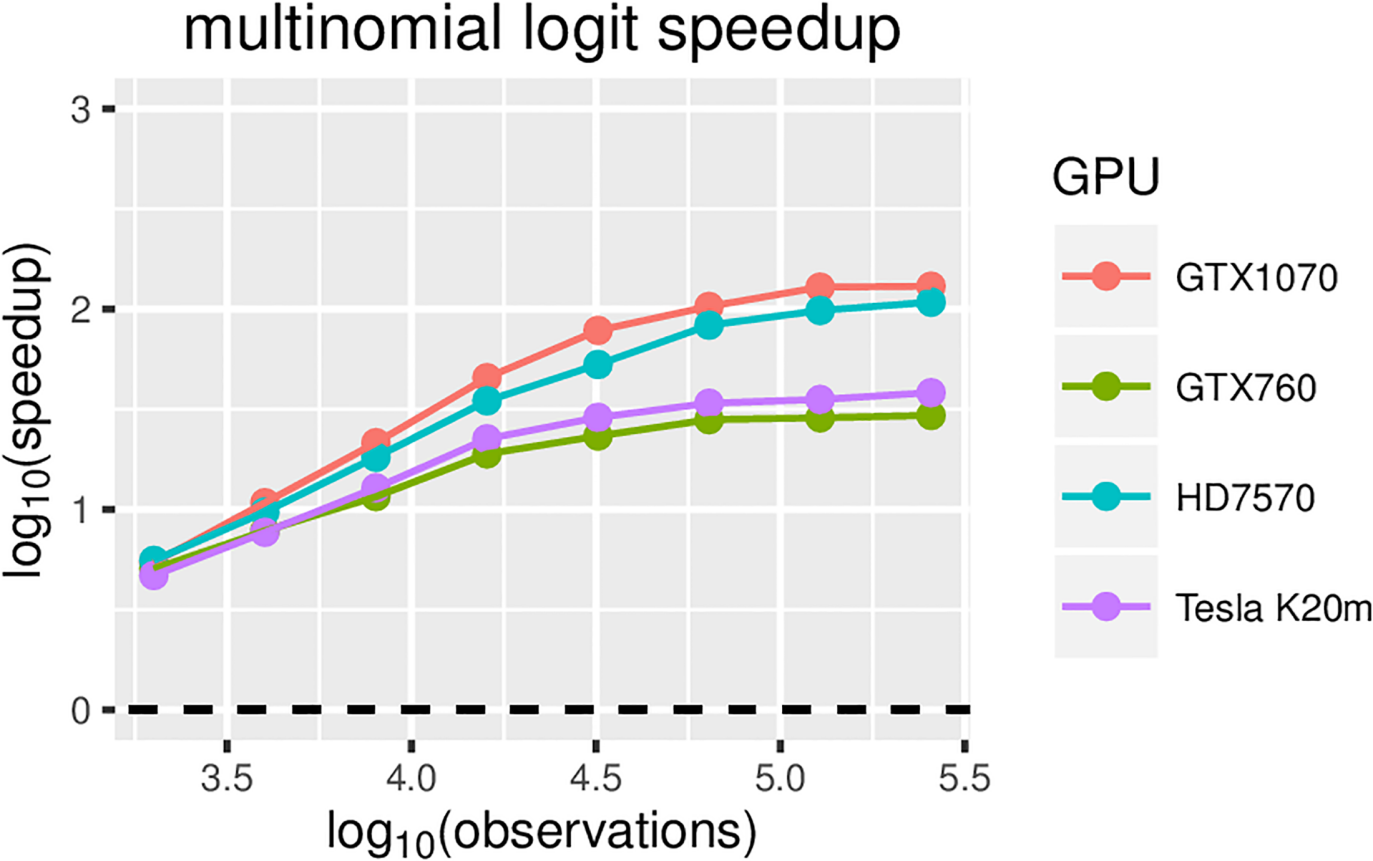
Multinomial logistic regression speedup relative to the CPU-only version (on an Intel i5-6600K @ 3.50 GHz) for different GPUs and an increasing number of observations.

**Fig 7:**
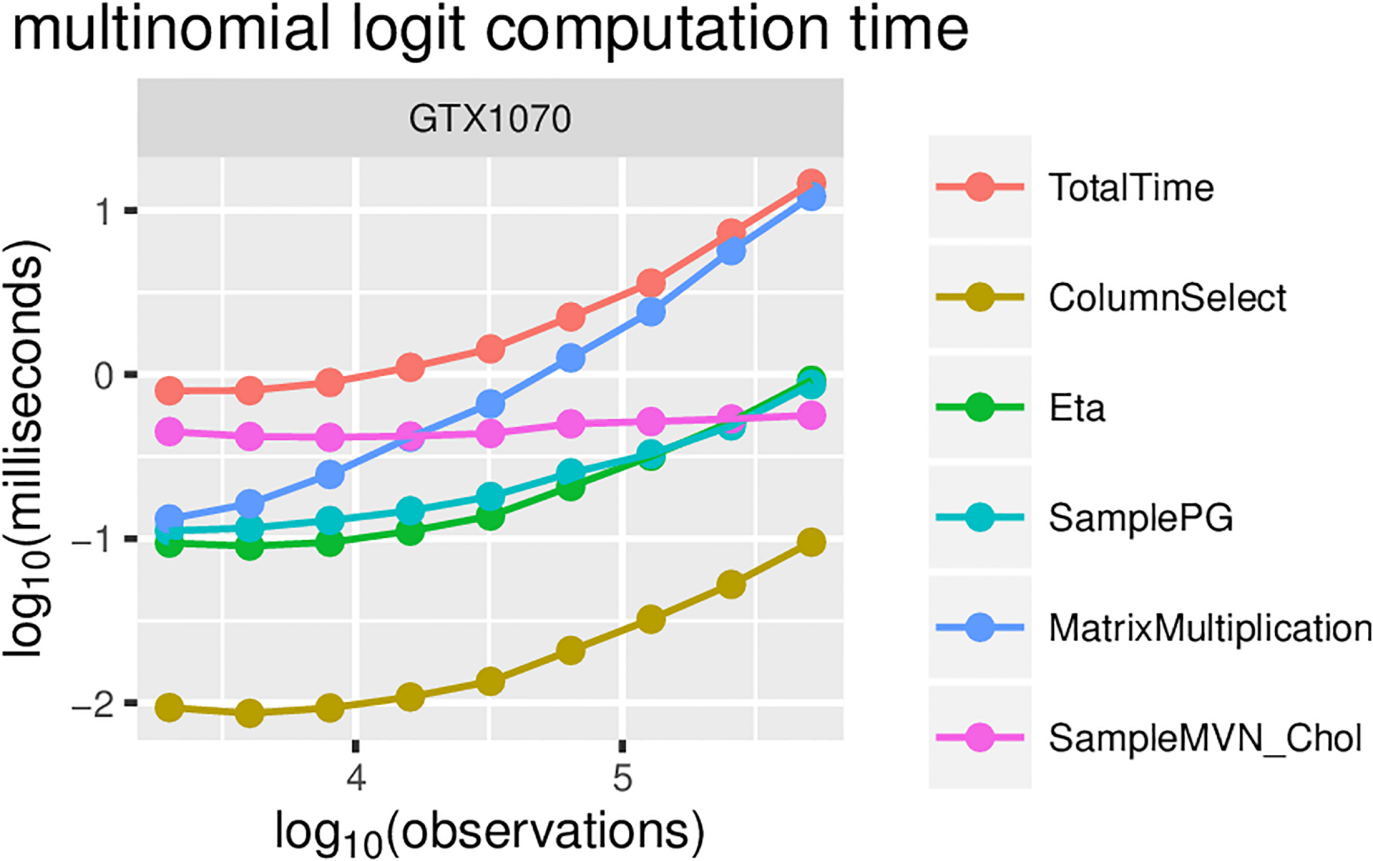
Multinomial logistic regression computation times for GTX1070, broken down by key algorithm components. The results on other GPUs are very similar, and thus, we omit them.

For multinomial regression, speedups of 100 fold are achieved for on the order of 10000 predictors (see Fig 6). This is also the maximum measured speedup achieved. With a fixed number of predictors, the computation under the GPU version is dominated by matrix multiplications, while the calculation of *η* and the sampling from PG each take approximately 10-times less time than matrix multiplication (see Fig 7).

**Fig 8:**
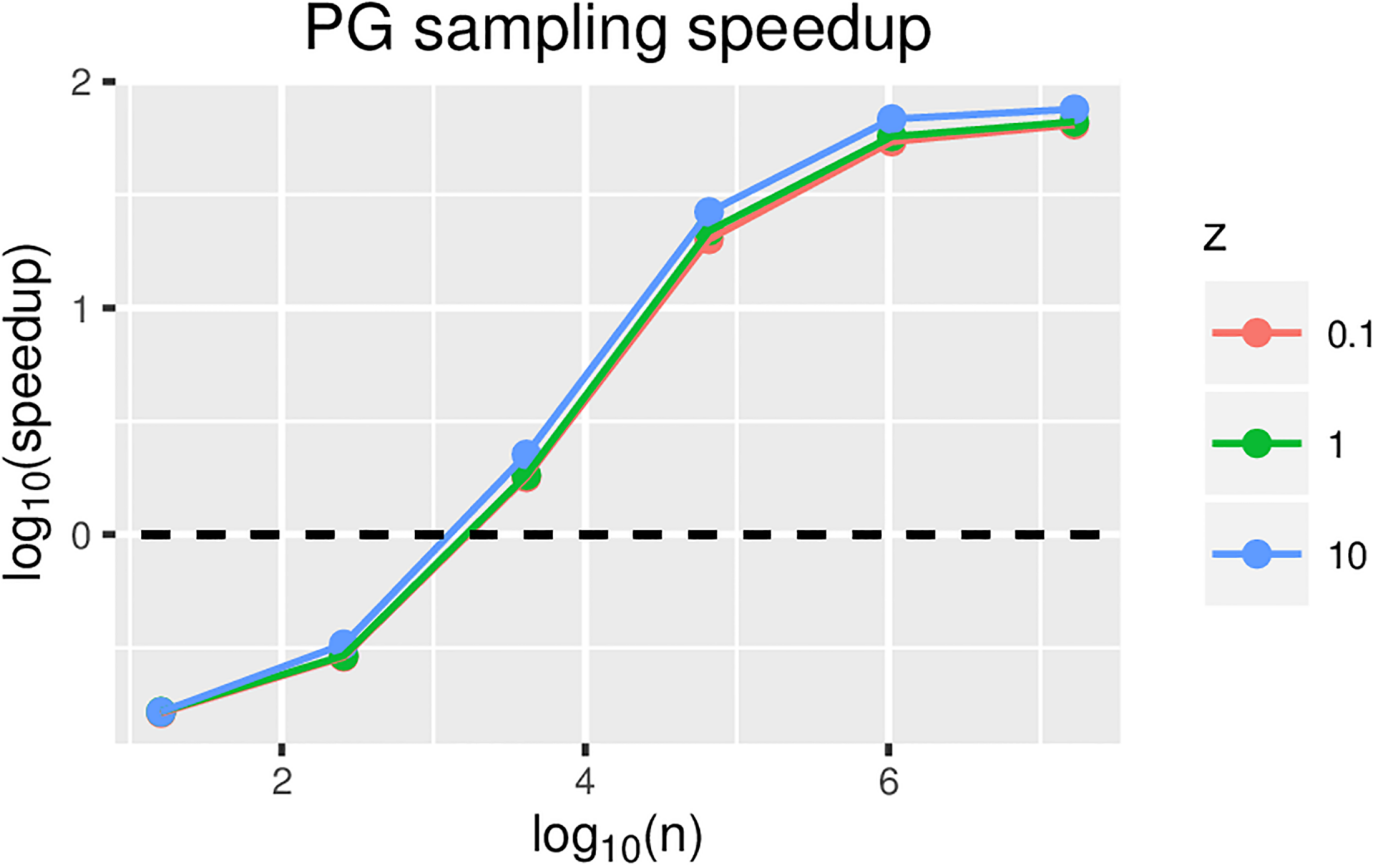
Polya-Gamma sampling speedup for GTX1070 relative to the CPU-only version (on an Intel i5-6600K @ 3.50 GHz) for increasing problem size and different values of *z*.

The parallelization of the sampling from the PG distribution remains practically relevant, however. When measured with more detail, the results suggest a speedup of approximately 50 fold for *n* on the order of 100,000 (up to a maximum of 100 fold when there are millions of observations) (see Fig 8). Therefore, the parallelization of the sampling from PG results in an overall speedup of approximately 5 fold with 100,000s of observations. The results also show that the speedup does not depend strongly on the value of *z*; however, larger values of *z* allow for a slightly better speedup. This is due to the higher probability of a more computationally complex execution path in PG sampling when *z* is larger.

Of the four GPUs used for the empirical evaluation, the Tesla K20m is the only compute-only GPU. However, the recency of the architectures of the other three GPUs outweighs this property in terms of overall performance. In Fig 6, we can see that the performance on the K20m is similar to on the GTX760, which is a more recent mid-range GPU. Because the K20m has a substantially larger number of computing cores, it outperforms the GTX760 for larger numbers of predictors in the Bayesian Lasso. As expected, the overall best performance is achieved by the most-recent high-end GPU of the four, the GTX1070. Given that the HD7570 is both older and a mid-to-high-end GPU, its performance is better than expected. This is mostly due to the large differences in its architecture compared to the other 3 GPUs. The architecture of the HD7570 is better suited for special cases whereby the number of threads is low, which is the case in the first steps of the Cholesky decomposition and the forward substitution in the matrix inversion.

## Conclusion

Our GPU implementations of Bayesian Lasso and multinomial logistic regression achieve 100-fold speedups on mid-level and high-end GPUs. Substantial speedups of 25 fold can also be achieved on older and lower end GPUs. Most of the speedup is due to the parallel matrix multiplications, which are the dominate computations as the problem grows; however, parallelizing the sampling from distributions, in particular, the PG distribution, also leads to substantial speedups. Because the samplers are implemented in OpenCL, they can be used on any type of GPU and other types of computational units, which is convenient in practice and advantageous compared to related work.

We have two main directions for future work. First, the tuning of the parameter values could be automated. Currently, default GPU implementation parameter values can be used or the user can set values manually. Default values still lead to substantial speedups but can result in an up to 50% drop in efficiency (relative to the optimal values). Therefore, the seamless auto-tuning of the parameters is of practical importance. We plan to exploit the iterative nature of Gibbs sampling (and MCMC in general), which lends itself to iterative parameter tuning because the matrix and vector dimensions, which have the largest effect on the optimal parameters, remain constant for all iterations. In addition, we plan to parallelize the general-purpose algorithms that are at the core of current state-of-the-art samplers for Bayesian inference, in particular, Hamiltonian Monte Carlo (HMC). Note that HMC mixes well even on imbalanced, large data sets, where Gibbs sampling has been shown to mix extremely poorly [29]. Additionally, we plan on implementing a GPU version of the QR decomposition with rank-p updates [30], which could, in some cases, be used as a less efficient but more numerically stable alternative to the Cholesky decomposition [7].

## Supporting information

S1 Data The data used in the empirical evaluation. The two data sets are stored as serialized R programming language objects (see saveRDS()). Each object holds the predictor matrix *X* and response vector or matrix *y* separately. A detailed description of the meaning of the predictors is not included because it is not relevant to this paper. Further information can be provided on request.
(RAR)

## References

[1] Suchard MA, Wang Q, Chan C, Frelinger J, Cron A, West M. Understanding GPU programming for statistical computation: Studies in massively parallel massive mixtures. Journal of Computational and Graphical Statistics. 2010;19(2):419–438. 10.1198/jcgs.2010.10016 20877443 PMC2945379

[2] da Silva AF. cudaBayesreg: Bayesian computation in CUDA. The R Journal. 2010;2(2):48–55.

[3] Takaishi T. GPU Computing in Bayesian Inference of Realized Stochastic Volatility Model. Journal of Physics: Conference Series. 2015;574(1):012143.

[4] Cossio P, Rohr D, Baruffa F, Rampp M, Lindenstruth V, Hummer G. BioEM: GPU-accelerated computing of Bayesian inference of electron microscopy images. Computer Physics Communications. 2017;210:163–171. 10.1016/j.cpc.2016.09.014

[5] Lee A, Yau C, Giles MB, Doucet A, Holmes CC. On the utility of graphics cards to perform massively parallel simulation of advanced Monte Carlo methods. Journal of computational and graphical statistics. 2010;19(4):769–789. 10.1198/jcgs.2010.10039 22003276 PMC3191530

[6] Yan F, Xu N, Qi Y. Parallel inference for latent dirichlet allocation on graphics processing units. In: Advances in Neural Information Processing Systems; 2009. p. 2134–2142.

[7] Terenin A, Dong S, Draper D. GPU-accelerated Gibbs Sampling. arXiv preprint arXiv:160804329. 2016;.

[8] Beam AL, Ghosh SK, Doyle J. Fast Hamiltonian Monte Carlo Using GPU Computing. Journal of Computational and Graphical Statistics. 2016;25(2):536–548. 10.1080/10618600.2015.1035724

[9] Friedman J, Hastie T, Tibshirani R. Regularization paths for generalized linear models via coordinate descent. Journal of statistical software. 2010;33(1):1 10.18637/jss.v033.i01 20808728 PMC2929880

[10] Tibshirani R. Regression shrinkage and selection via the Lasso. Journal of the Royal Statistical Society Series B (Methodological). 1996; p. 267–288.

[11] Park T, Casella G. The Bayesian Lasso. Journal of the American Statistical Association. 2008;103(482):681–686. 10.1198/016214508000000337

[12] Gramacy RB. monomvn: Estimation for Multivariate Normal and Student-t Data with Monotone Missingness; 2016. Available from: http://CRAN.R-project.org/package=monomvn.

[13] Bishop CM. Pattern Recognition and Machine Learning. Springer-Verlag New York; 2006.

[14] Polson NG, Scott JG, Windle J. Bayesian inference for logistic models using Pólya–Gamma latent variables. Journal of the American statistical Association. 2013;108(504):1339–1349. 10.1080/01621459.2013.829001

[15] Polson NG, Scott JG, Windle J. Bayesian inference for logistic models using Polya-Gamma latent variables; 2013. Available from: http://arxiv.org/abs/1205.0310.

[16] Devroye L. On exact simulation algorithms for some distributions related to Jacobi theta functions. Statistics & Probability Letters. 2009;79(21):2251–2259. 10.1016/j.spl.2009.07.028

[17] Luc D. Non-uniform random variate generation. NY: Springer 1986;.

[18] Windle J, Polson NG, Scott JG. Sampling Polya-Gamma random variates: alternate and approximate techniques. arXiv preprint arXiv:14050506. 2014;.

[19] NVIDIA Corporation. NVIDIA CUDA Compute Unified Device Architecture Programming Guide. NVIDIA Corporation; 2007.

[20] Tan G, Li L, Triechle S, Phillips E, Bao Y, Sun N. Fast Implementation of DGEMM on Fermi GPU. In: Proceedings of 2011 International Conference for High Performance Computing, Networking, Storage and Analysis. SC’11. New York, NY, USA: ACM; 2011. p. 35:1–35:11. Available from: http://doi.acm.org/10.1145/2063384.2063431.

[21] Lai J, Seznec A. Performance upper bound analysis and optimization of SGEMM on Fermi and Kepler GPUs. In: Proceedings of the 2013 IEEE/ACM International Symposium on Code Generation and Optimization (CGO); 2013. p. 1–10.

[22] Louter-Nool M. Block-Cholesky for Parallel Processing. Appl Numer Math. 1992;10(1):37–57. 10.1016/0168-9274(92)90054-H

[23] Mahfoudhi R, Mahjoub Z, Nasri W. Parallel communication-free algorithm for triangular matrix inversion on heterogenoues platform. In: 2012 Federated Conference on Computer Science and Information Systems (FedCSIS); 2012. p. 553–560.

[24] Marsaglia G. Xorshift RNGs. Journal of Statistical Software. 2003;8(1):1–6.

[25] Demchik V, Kolomoyets N. QCDGPU: Open-Source Package for Multi-GPU Monte Carlo Lattice Simulations. Computer Science. 2014;1(1):13–21.

[26] Manssen M, Weigel M, Hartmann AK. Random number generators for massively parallel simulations on GPU. The European Physical Journal Special Topics. 2012;210(1):53–71. 10.1140/epjst/e2012-01637-8

[27] Box GEP, Muller ME. A Note on the Generation of Random Normal Deviates. Ann Math Statist. 1958;29(2):610–611. 10.1214/aoms/1177706645

[28] Mersmann O. microbenchmark: Accurate Timing Functions; 2015. Available from: http://CRAN.R-project.org/package=microbenchmark.

[29] Johndrow JE, Smith A, Pillai N, Dunson DB. Inefficiency of Data Augmentation for Large Sample Imbalanced Data. arXiv preprint arXiv:1605.05798.

[30] Andrew R, Dingle N. Implementing QR factorization updating algorithms on GPUs. Parallel Computing. 2014; 40(7):161–172. 10.1016/j.parco.2014.03.003

